# Carrying out an optimal experiment

**DOI:** 10.1107/S0907444909038578

**Published:** 2010-03-24

**Authors:** Zbigniew Dauter

**Affiliations:** a Synchrotron Radiation Research Section, MCL, National Cancer Institute, Argonne National Laboratory, Argonne, IL 60439, USA

**Keywords:** diffraction data collection, data-collection strategies, diffraction experiments

## Abstract

Diffraction data collection is the last experimental stage in structural crystallography. It has several technical and theor­etical aspects and a compromise usually has to be found between various parameters in order to achieve optimal data quality. The influence and importance of various experimental parameters and their consequences are discussed in the context of different data applications, such as molecular replacement, anomalous phasing, high-resolution refinement or searching for ligands.

## Abstract 2

Diffraction data collection parameters leading to optimal data quality are discussed in the context of different applications of these data.

## 1. Introduction

Diffraction data collection is the last truly experimental step in the process of solving a macromolecular crystal structure. The subsequent stages of the process are mostly computational and can easily be repeated or modified. Good-quality data will always make structure solution easier and will produce more faithful electron density as well as a more accurate atomic model. It is therefore important to carry out the diffraction experiment under optimal conditions. Often, simple mistakes during data collection result in much time being wasted in unsuccessful attempts to solve the structure; on the other hand, the short time required for optimizing the procedure can lead to rapid and successful finalization of the project.

Defining an ‘optimal diffraction experiment’ unequivocally, however, is not easy. There are several criteria for data quality and these criteria may have different relative weights or priorities depending on the particular application. Among these criteria, the most important are completeness, accuracy and maximum resolution. Naturally, it is ideal to have, at the end of the data-collection session, a 100% complete data set of highly accurate reflection intensities extending to ultrahigh resolution. In the real world, however, it is rarely possible to achieve these goals simultaneously because of limitations resulting from the crystal characteristics, time restrictions, the properties of the available X-ray source and detector, and the particulars of the hardware and software. Data collection always involves finding a compromise between these limitations, and optimization of the whole experiment requires proper weighting of the various criteria. Putting too much weight on data completeness and multiplicity may result in poor data accuracy owing to radiation damage; an excessive tendency to avoid radiation damage may result in underexposed diffraction images and limited resolution *etc.* The optimal experiment, therefore, requires a wise compromise between various competing requirements.

## 2. Various requirements and applications

Different applications require that various characteristics of diffraction data have different levels of importance and that special attention be directed to those particular aspects of data quality during the experiment (Table 1 ▶). The various intended applications include molecular replacement, anomalous diffraction phasing, atomic model refinement, searching for bound ligands and some less frequently used types of experiment.

*Molecular replacement* is based on the comparison and superposition of two Patterson syntheses, the first calculated from an existing search model and the second computed from the measured diffraction data (Dodson, 2008 ▶). Since Patterson synthesis utilizes squares of reflection amplitudes, the strongest reflections are especially important. Because the rotation and translation functions are computed at relatively low resolution (usually less than 3 Å), the diffraction data do not need to extend too far in resolution. It is important that all strong low-resolution ranges are complete (Davies, 1993 ▶). The data accuracy is of secondary importance, but if strong reflections are missing they in fact contribute to Fourier syntheses with zero amplitude, strongly biasing the appearance of the electron density or Patterson map. For this application, the highest priority is the completeness of the low-resolution reflections.

*Anomalous diffraction phasing* utilizes small differences between the intensities of Friedel-related reflections. In typical multiple- or single-wavelength anomalous diffraction (MAD or SAD; Hendrickson & Ogata, 1997 ▶; Dodson, 2003 ▶) cases the Bijvoet ratio Δ*F* ^±^/*F* is between 3% and 6% and in sulfur-SAD it may be smaller than 1%. To ensure that the resulting anomalous signal is significant, the accuracy of the measured intensities has to be very high, of the order of a few percent. Thus, small differences can easily be overwhelmed by the effects of radiation damage and care should be taken not to overexpose the diffraction images. The location of anomalous sites by Patterson or direct methods requires that the strongest low-resolution reflections are complete (Vekhter, 2005 ▶). The anomalous data should therefore be characterized by high accuracy and completeness, but do not need to extend to the full resolution potential of the crystal. It is safer to solve the structure from a modestly exposed accurate data set and then refine the model against separate (possibly native) high-resolution data.

*Atomic model refinement* should be performed against as high a resolution as the crystal can provide (Tronrud, 2004 ▶). Having a complete and accurate data set is preferable, but refinement is possible even when these two criteria are not fully satisfied. However, a lack of strong reflections owing to overloaded detector pixels may cause severe bias of the electron-density maps and lead to the misinterpretation of fine structural features. Sometimes, performing multiple passes of data collection is necessary: firstly at limited resolution and modest exposure, avoiding overloads, and subsequently with longer exposures and the full extension of resolution, permitting the strongest reflections to be overloaded. The low-resolution pass should be performed first, when the crystal is not radiation-damaged. All measured intensities should then be scaled and merged together into one final data set.

*Searching for bound ligands*, often performed by pharmaceutical companies, requires many data sets to be collected quickly but not necessarily highly accurately (Kleywegt, 2007 ▶). After preliminary inspection, a comprehensive data set can be measured later on the selected crystal. The priority for the initial search is given to speed and possibly the automation of crystal mounting and the data-collection process, with less weight placed on other quality criteria.

An important type of data-collection experiment is the *measurement of data from crystals of large structures*, such as multi-protein or protein–nucleic acid complexes, which are often only able to provide a few exposures before deteriorating from radiation damage. In such cases, data have to be collected from many crystals, with intensities merged from the set of the most isomorphous specimens. While it is difficult to obtain accurate and complete data in this manner, such data may lead to significant biological discoveries (Harrison, 2004 ▶). This type of experiment requires an enormous amount of patience from the people conducting the project. The automated mounting of a large number of crystals may be very beneficial, but it is better to govern data collection with a human, not a robot, since each crystal has to be carefully evaluated individually.

Sometimes data may be measured for *structure solution by direct methods* (Usón & Sheldrick, 1999 ▶). In this case, one should measure reflections extending to as high a resolution as possible and even relax the usual quality criteria. A small fraction of meaningful intensities among the majority of ‘unobserved’ ones measured beyond 1.2 Å resolution may lead to successful structure solution. Of course, overall data have to be complete, especially at the lowest resolution.

## 3. Choice of data-collection parameters

To perform diffraction data collection by the rotation method (Arndt & Wonacott, 1977 ▶), appropriate experimental parameters, such as radiation wavelength, crystal rotation range and rotation start and possibly orientation, exposure time and/or beam attenuation, crystal-to-detector distance, beam size *etc.*, have to be selected. Discussion of the influence of these parameters is available in several publications (Dauter, 1999 ▶, 2005 ▶; Mitchell *et al.*, 1999 ▶) together with various illustrative figures relevant to the aspects discussed below.

In the context of *data completeness*, the most important role is played by the total rotation range and the rotation start position. These in turn depend on the crystal symmetry and its orientation on the goniostat. In principle, the minimum rotation range necessary is that covering the complete asymmetric unit of reciprocal space in the case of native data or two such units related by the symmetry center or mirror plane in the case of anomalous data. The asymmetric unit is always wedge-shaped, with its apex at the origin, and is limited by the resolution sphere and the mirror planes of the Laue symmetry group. For example, for a *P*1 crystal native data require the hemisphere of reciprocal space to be covered; for anomalous data all the reflections in the entire reciprocal sphere have to be measured. For a crystal of the 622 class, both native and anomalous data require 30° of total rotation around the *c* axis and 90° of total rotation around the axis lying in the *ab* plane. Moreover, in the second example the starting point of rotation has to correspond to the crystal orientation at which its sym­metry axes are either parallel or perpendicular to the beam direction. To achieve data completeness, an arbitrary crystal orientation would require an intermediate amount of rotation and in practice it is best to formulate the appropriate strategy using one of the existing strategy programs, which are run after interpreting one or two preliminary diffraction images (Popov & Bourenkov, 2003 ▶; Bourenkov & Popov, 2006 ▶).

It should be noted that the above reasoning gives the minimum rotation range necessary for data completeness. However, it may be advantageous to cover more than the minimum rotation, assuming the effects of radiation damage do not spoil the benefits of the increased multiplicity of measurements (Ravelli & Garman, 2006 ▶).

The amount of crystal *rotation per image* should be adjusted to avoid excessive overlap of reflection profiles. In the ‘wide-slicing’ mode, diminishing the image width below the value of the rocking curve (the sum of the crystal mosaicity and beam divergence) does not provide the additional benefit of lowering the background. However, the ‘fine-slicing’ mode, with Δϕ less than or equal to 0.1°, when a reflection is present on a series of consecutive images, is beneficial for detectors with low dead-time because it enables the construction of more accurate three-dimensional profiles of each measured reflection (Pflugrath, 1999 ▶).

The selection of radiation *wavelength* (at synchrotron beamlines) depends on the intended application. If the data are being measured for a MAD experiment, the selected wavelengths have to be adjusted to the absorption edge of the appropriate element on the basis of the recorded fluorescence spectra. For SAD data the wavelength may be at the high-energy remote region of the anomalous scatterers’ spectra or within the range 1.7–2.2 Å if the anomalous signal comes from elements having no easily accessible edges (P, S, K, Ca, I, Xe, Cs). For the native data, there is no strong preference for the wavelength selection; usually, around 1 Å is a good choice that corresponds to the most intense region of the X-ray beam at a typical macromolecular crystallography beamline. At home laboratories one is usually confined to the copper-source wavelength of 1.54 Å, although chromium anodes with λ = 2.23 Å are also available for light-atom SAD work (Yang *et al.*, 2003 ▶).

The *crystal-to-detector distance* should be adjusted such that the entire area of the detector is used to record data. Too close a distance leaves the outer areas of the detector unused. When this occurs, the noise level is higher than necessary because the background intensity diminishes with the square of the distance, whereas the reflection profiles usually do not increase much as the crystal-to-detector distance is increased. In practice, it is good to judge by eye how far out reflections are visible at the highest display contrast and then set the detector distance to a maximum resolution about 0.2 Å higher than this limit. Of course, eventually the final resolution limit should be decided after data scaling and merging to extend to an average *I*/σ(*I*) value of about 2.0. If one of the crystal cell dimensions (that in the plane of the detector) is large, the increased detector distance will increase the inter-spot distances and make the integration of intensities easier. However, increasing the detector distance does not help if the spot overlap results from a very long cell dimension parallel to the X-ray beam. When one unit-cell parameter is much longer than the other two, it is beneficial to orient it to be more or less parallel to the goniostat spindle axis; this way, it will never be parallel to the X-ray beam. This can be achieved by using a kappa goniostat or an appropriately bent cryo-loop.

The appropriate *exposure time* should be adjusted after a couple of initial exposures. There should be no, or just a very few, overloaded pixels visible on the displayed diffraction images. If appropriate measurement of weak high-resolution reflections requires long exposures displaying many overloads, data must be collected in multiple passes, first by covering the low resolution with short exposures or an attenuated beam and then in the next pass aiming at the weak highest resolution data. Intensities from all passes should then be scaled and merged. For successful scaling, it is advisable not to exceed a difference of more than tenfold in the effective exposure between consecutive passes. At some synchrotron beamlines, the synchronization of the spindle motor with the X-ray shutter may not be ideal and it may be unsafe to collect data faster than, say, 1° per second; if necessary, the beam should be attenuated with metal foils.

## 4. Conclusions

Diffraction data collection from macromolecular crystals, particularly at synchrotron beamlines, involves many technical points, but in spite of the use of automation and robotics it remains a scientific process. It is finally the responsibility of the experimenter, not of the robot, to ensure that the diffraction data are measured optimally. This requires the correct adjustment of a large number of parameters and finding an optimal compromise between several factors. Existing strategy programs can help in some aspects, but to achieve the ultimate data quality it is always advisable to engage a human brain in the decision-making process.

## Figures and Tables

**Table 1:** Relative importance of various aspects of data collection in different applications The priorities of different aspects of data are graded from very high (++++) to not very important (+).

	Molecular replacement	Anomalous phasing	High-resolution refinement	Ligand search
Accuracy	+	++++	++	++
Low-resolution completeness	+++	+++	++	++
Resolution	+	+	+++	++
Overall completeness	++	++	++	++
Automation	++	+	++	+++
